# PFOh No: Spontaneous Globe Rupture During Injection of Perfluoro-*n*-Octane (PFO) During Retinal Detachment Repair

**DOI:** 10.3389/fmed.2020.582596

**Published:** 2020-10-23

**Authors:** Jon David Hammer, Syed Gibran Khurshid

**Affiliations:** Department of Ophthalmology, University of Florida, Gainesville, FL, United States

**Keywords:** perfluoro-n-octane, PFO, retina, retinal detachment (RD), globe rupture

## Abstract

The authors describe a case of spontaneous globe rupture during instillation of perfluoro-*n*-octane (PFO) during retinal surgery. A 71-year-old male with a macular-involving rhegmatogenous retinal detachment underwent pars plana vitrectomy. During instillation of PFO manually on a single-bore cannula by the assisting resident, spontaneous globe rupture occurred superotemporally in an area with no visible underlying structural abnormalities. Factors that led to this complication include the use of single-bore cannula, error in judgment of resistance during PFO injection, and inexperience. This is the first report of this complication without an identifiable structural abnormality predisposing patient to perforation.

## Introduction

Perfluoro-*n*-octane (PFO) is a perfluorocarbon liquid (PFCL) used in retina surgery to aid in the aspiration of subretinal fluid (SRF) and stabilization of the retina during vitrectomy due to its characteristics including low viscosity, high density, transparency, and clarity (1, 2). PFCLs are considered safe and effective adjuvant tools for the repair of retinal detachments mainly intraoperatively, and they are completely removed at the end of the case due to concerns for retained PFCLs causing an inflammatory reaction and potential toxicity (3, 4). One concern related to the use of PFO intraoperatively is the acute rise in intraocular pressure (IOP) during instillation, which can cause complications if not appropriately controlled. We present a case of spontaneous globe rupture during the instillation of PFO during retinal detachment surgery related to an acute rise in IOP and include a literature review of this rare complication.

## Case Report

A 71-year-old male presented with 3 weeks of decreased vision in his right eye (oculus dexter [OD]). The patient had a history of uncomplicated cataract surgery in both eyes (oculus uterque [OU]) 5 years prior, mild myopia OU (spherical equivalent −1.50 OU), and no history of ocular trauma. His vision was 20/400 OD and 20/20 in the left eye (oculus sinister [OS]). Intraocular pressure (IOP) measured 8 mmHg OD and 11 mmHg OS. Dilated fundus exam revealed temporal macular-involving rhegmatogenous retinal detachment OD with horseshoe tear at 7 o'clock and no retinal abnormalities OS. There was no ectasia or staphyloma noted on this exam or on prior exams.

The patient underwent pars plana vitrectomy for retinal detachment repair, assisted by a junior resident under retrobulbar block. Surgery began by performing a three-port pars plana vitrectomy using 23-gauge valved trocars and a vitrectomy system with an integrated pressurized infusion system that includes an electronic feedback-controlled IOP system (5). Next, to aid in draining the subretinal fluid, PFO was manually administered via a single-bore 23-gauge cannula on a syringe operated by the junior resident sitting temporally while the attending surgeon controlled the single-bore cannula in the superotemporal trocar and kept the light pipe in the superonasal trocar to maintain direct visualization of the retina. When the junior resident experienced resistance with the PFO injection, he was supposed to pause instillation and alert the attending surgeon, who would then manually release pressure via the superonasal trocar by intermittently removing the light pipe and engaging the valve. Once pressure was released, the light pipe would be reinserted and the junior resident would then resume PFO instillation. The retina was visualized during active PFO instillation and there was no blanching of retinal vessels noted by the surgeon. After ~2 cc of PFO had been placed, the vitreous cavity suddenly filled with bubbles. Upon further inspection of the eye, intense chemosis was noted. A temporal peritomy was then performed and a superotemporal scleral perforation ~3 mm in length was noted behind the rectus muscle insertion. The perforation was then closed using interrupted 7–0 vicryl sutures. Watertight sealing of the perforation was confirmed. Then, we proceeded with a retinotomy to relieve retinal incarceration, fluid–air exchange, endolaser, and silicone oil tamponade. On post-op day 1, the patient had visual acuity (VA) 20/200 OD, IOP 9 mmHg, and an attached retina under oil. At 5 months the silicone oil was surgically removed, and at 11 months the retina remained attached with VA 20/200 OD.

## Discussion/Conclusion

Spontaneous globe rupture during PFO instillation is a very rare complication of PFO placement. To our knowledge, this is the first report of this complication without an identifiable predisposing structural scleral abnormality.

Diamint et al. reported a case with spontaneous globe rupture during PFO instillation via vitrectomy probe, but that patient was found to have an area of severe scleromalacia at the rupture site (6). Also, unlike our case, that patient developed a large subretinal hemorrhage, subconjunctival hemorrhage, and significant emphysema of the orbit, face, and neck. Three other cases have been reported in the literature of the development of emphysema after PFO instillation, but none of these were thought to be directly due to a PFO-related injury. Two occurred in the setting of a perforating foreign body injury (7, 8). The third was thought to be related to an occult injury by the retrobulbar needle, but the authors were unable to determine during which step of the surgery did the perforation occur and could not rule out rupture of an unidentified staphyloma or spontaneous ocular explosion (9).

In our case, it was clear that rupture occurred during PFO instillation. The factors that led to this complication include the use of a single-bore cannula, error in judgment of resistance during PFO injection, and inexperience.

**Figure 1:**
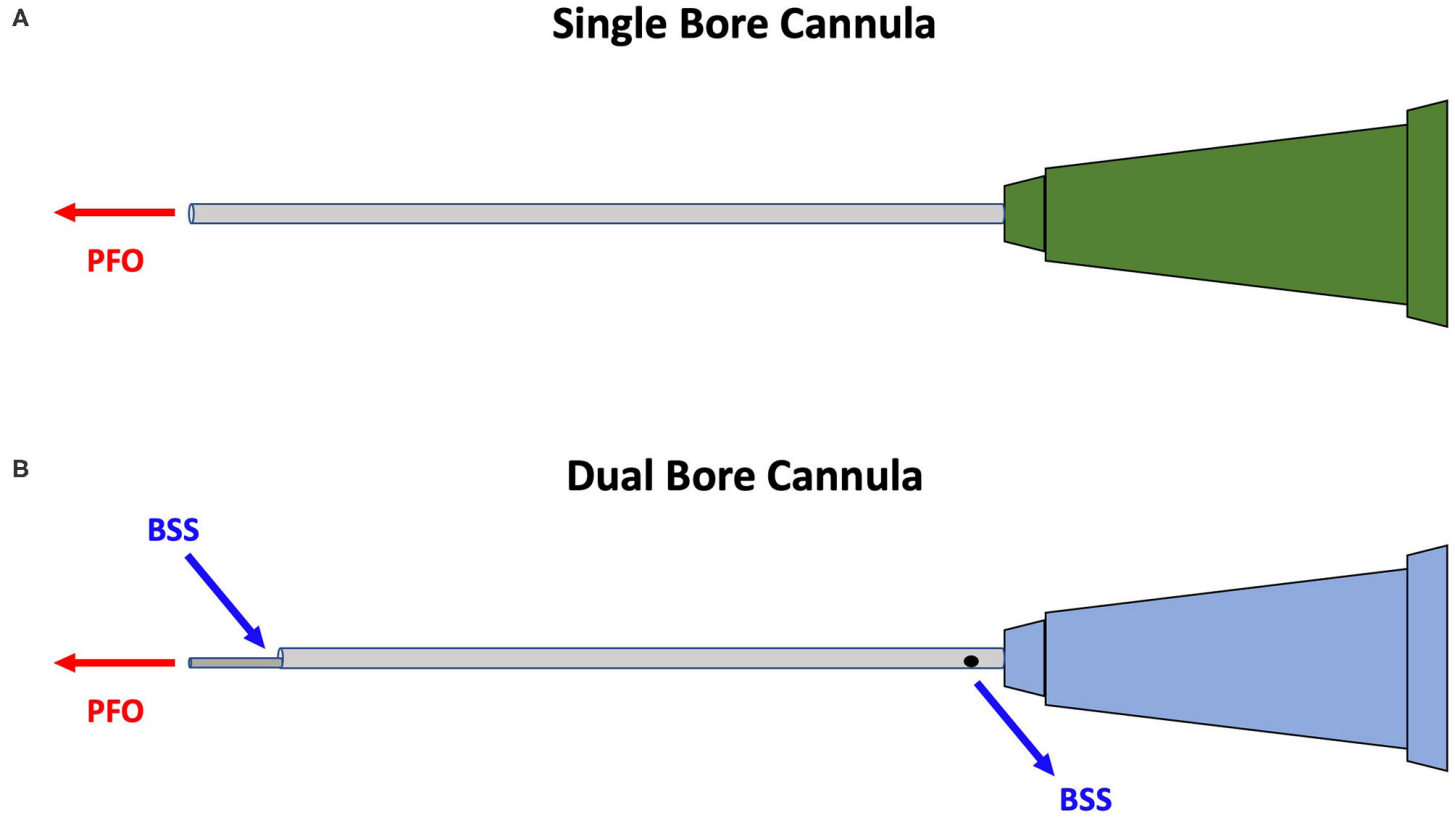
Illustration showing that the single-bore cannula **(A)** allows instillation of perfluoro-*n*-octane (PFO) alone while the dual-bore cannula **(B)** allows passive egress of balanced saline solution (BSS) in addition to the instillation of PFO in order to maintain pressure equilibrium. Digital illustration created by author Jon David Hammer, with permission given to include in the publication of case report.

The single-bore cannula used in this case allows the passage of fluid through one lumen. A dual-bore cannula may have prevented this complication by also allowing passive egress of fluid through a secondary lumen to maintain pressure equilibrium (Figure 1). Our academic institution now exclusively uses dual-bore cannulas.

The vitrectomy system used in our case maintains IOP via an electronic feedback system that involves setting a desired IOP and using sensors to monitor the aspiration and irrigation flow rates and adjusts the irrigation rates accordingly to maintain fluid balance (5). In our case, however, this feedback system could not prevent a large spike in IOP because the PFO was being manually instilled on a cannula while aspiration was also performed manually. The infusion flow rate can decrease, but not actively aspirate. When using the manual technique described in this case, it is necessary to pause PFO instillation when resistance is met and allow aspiration to equilibrate pressure before continuing. In our case, there was an error in judgment of resistance and PFO continued to be injected without adequate aspiration.

The error in judgment of resistance is directly related to the third factor: inexperience of the first-year ophthalmology resident, who was assisting his first retina case. Therefore, it is important for residents to have increased hands-on wet lab experience and to be prepared for all surgical cases.

For a globe rupture to occur from the instillation of fluid in a visibly normal eye, IOP was measured to reach 817–6,403 mmHg with an average of 1.9 ml of injected saline in human cadaver and eye bank eyes, according to one study by Bullock et al. (10). Approximately 2 cc of PFO was instilled in our patient, so either IOP reached the range above or an underlying occult weakness led to the rupture at a lower pressure. We can rule out injury from the retrobulbar needle since it was administered in a different quadrant from the rupture.

In summary, spontaneous globe rupture is a rare complication that can occur during the instillation of PFO during retina surgery, even without an identifiable structural abnormality. Precautions that could prevent this complication in the future include the use of dual-bore cannulas for PFO instillation and more hands-on wet lab experience for junior residents.

## Data Availability Statement

The original contributions presented in the study are included in the article/supplementary material, further inquiries can be directed to the corresponding author/s.

## Ethics Statement

Written informed consent was obtained from the individual(s) for the publication of any potentially identifiable images or data included in this article.

## Author Contributions

JH and SK performed the surgery describe in the case report. SK conceived the idea of describing the case report and reviewed, made significant edits, and revisions to the manuscript. JH performed the literature review and was a major contributor in writing the manuscript and designed the figure included. Both authors read and approved the final manuscript.

## Conflict of Interest

The authors declare that the research was conducted in the absence of any commercial or financial relationships that could be construed as a potential conflict of interest.
